# 

**Published:** 1892-08-06

**Authors:** 


**Notices of Births, Deaths, and Marriages,** are inserted in this column at a charge of 2s. 6d. for an announcement not exceeding four lines.

### NOTICE TO CORRESPONDENTS.

All MS., Letters, Books for Review, and other matters intended for the Editor should be addressed The Editor, The Lodge, Porchester Square, London, W.

The Editor cannot undertake to return rejected M.S., even when accompanied by stamped directed envelope.

**Notice to Advertisers and Subscribers.**

All Advertisements, Orders for Copies of The Hospital, and Business Communications should be addressed to The Publisher, *not to the Editor*, The Hospital, 140, Strand, W.C.

The Cost of Subscription, post free, is as follows:—Per week, 2½d.; per quarter, 2s. 6d.; per half-year, 5s.; per annum, 8s. 6d. Foreign:—Per annum, 10s. 8d.; payable, in all cases, in advance.

**"Burdett's Hospital Annual," 1891—1892.**

Third year of publication. 600 pp. Boards, gilt lettering. A most complete guide to British and Colonial Hospitals, Dispensaries, Nursing and Convalescent Institutions, and Asylums. Price 3s. 6d. Published by The Scientific Press (Limited), 140, Strand, W.C.

**NOTICE.—The Index for Vol. XI. (ending March, 1892) is on sale at the Office of this Journal, price 2½d., post free.**

### Scraps and Gleanings.

The total amount collected by the ladies of Hull on Hospital Saturday was £537 19s. 8d.

The Queen has approved the appointment of Mr. George A. Stevenson to be a Commissioner of Public Works in Ireland.

It is reported that the marriage of Princess Marie of Edinburgh and the Crown Prince of Roumania will take place early in August.

The Court of Common Council have appointed Mr. Sims Reeves a professor of solo singing at the Guildhall School of Music at a fee of 30s. per hour.

Her Royal Highness the Duchess of Albany distributed the prizes to the successful competitors at the annual show of the Esher Cottage Garden Society, held at Sandown Park.

The Duchess of Teck laid the corner-stone of the new Church of the Good Shepherd, Mansfield Road, Gospel Oak. A guard of honour was furnished by the 17th Middlesex Rifle Volunteers.

The Worshipful Company of Goldsmiths have sent £100 to the Children's Country Holidays Fund (10, Buckingham Street, Strand, W.C.), of which the Hon. Alfred Lyttelton is the treasurer.

The Executive Committee of the Birmingham Hospital Saturday Fund report that this year's collection amounts to £11,908 3s. Of this sum £11,277 0s. 1d. was collected in industrial establishments.

At the end of last week the Hospital Saturday Fund amounted to near £10,000, of which £4,000 has been paid into the central office from district committees, as the result of the Ladies' Street Collection.

The Princess of Wales has forwarded a cheque for £150 to the British Home for Incurables, being the second instalment on account of the sale of Canon Fleming's sermon, "Recognition in Eternity."

The Lord Mayor has received a donation of £1,000 from Mr. Robert Davies, of Bodlondeb, Bangor, towards the Mansion House Fund for the relief of the sufferers by the recent fire at St. John's, Newfoundland.

During the past year 602 in-patients, and 1,000 out-patients, were treated at the Hartlepools Hospital, making in all 229 more patients treated than in the previous year. The working men of the Hartlepools contributed £577 to the hospital.

The expenditure of the Weymouth and Dorset County Royal Eye Infirmary for the past year amounted to £1,093 5s. 11d., and the receipts to £1,055 12s. 6d., leaving a balance due to Treasurer of £37 13s. 5d. There was a slight increase among the patients during the year.

Mr. James Cameron, the medical officer of health for Hendon, met with a fatal accident at Mill Hill. He was driving in his brougham, when the horse took fright and bolted. Dr. Cameron jumped out, and fell heavily on his head, fracturing his skull.

At the annual meeting of the Wakefield Clayton Hospital, the President said he thought that as they were likely to have an increase to the endowment ere long, they might furnish an additional ward. A much-needed requirement has been added to the hospital by the erection of two lifts.

An old man who died a few weeks ago at Berlin was discovered to have kept a record of his life in a book, noting, among other items, that he had smoked 628,713 cigars, worn out 298 shirts, and 326 collars, and drunk 28,786 glasses of Bavarian beer, and 33,081 glasses of Cognac and other spirits.

Professor Fresenius, one of the late Baron Liebig's most eminent pupils, and one of the foremost living chemists in Germany, last Friday celebrated the jubilee of his graduation as Doctor of Philosophy. He was on this occasion presented with the freedom of the City of Wiesbaden.

Two ladies who, in 1849, served as common soldiers in the revolutionary army, and fought in several of the fiercest battles, died recently in Hungary. One of them was several times promoted, and attained the rank of first lieutenant of Hussars. The other was decorated for valour in the field.

The Sixty-fourth Session of the Army Medical School, Royal Victoria Hospital, Netley, was brought to a close with the distribution of prizes by Colonel Hanning Lee. For the British Medical Service twenty-five candidates were successful; and for the Indian Medical Service seventeen passed.

The convalescent home given by Mr. Passmore Edwards to his native county, Cornwall was formally opened, a few days ago, by Mrs. Edwards. It is situated at Perranporth, about nine miles from Truro, on the north coast. Mr. Edwards has given £3,000 towards its endowment, and handed it over to the governor of the Royal Cornwall Infirmary, Truro.

The new sanatorium at Cardiff affords accommodation for 24 patients. The main ward pavilion, which is a one-storey building, consists of central administration block, containing Matron's sitting-room, bedrooms, kitchen, scullery. The two wards which terminate the main corridor are for males and females respectively. At the end of each ward is a bath-room, with portable baths on wheels.

During Tuesday night of last week a series of severe earthquake shocks was experienced at Pentewan, near Mevagissey, Cornwall. Many of the inhabitants left their beds and flocked into the street. Three more shocks occurred with brief intervals. The ground trembled violently, walls vibrated, and ornaments were knocked down. Rumbling noises preceded the earthquake shocks, and the night was intensely hot.

The office of Surgeon to the Queen in Ireland has been offered to and accepted by Sir William Stokes. The vacancy was caused by the death of Mr. William Colles, and the appointment of Sir William Stokes will receive hearty approval as being well merited. He has been throughout his life an untiring worker at surgery, and his writings, at once graceful and solid, have made his name familiar to all who are readers of the literature of the profession.

The Lady Mayoress has handed over a cheque for £1,028 9s. 9d. to Mr. Edwin Lawrence, and Mr. R. G. Kestin, the Secretary of the Royal Hospital for Women and Children, being the net profits of the Rose Show recently held at the Mansion House in aid of that institution. The committee have presented the Lady Mayoress with an illuminated address of thanks enclosed in a handsome album, and elected her an honorary life governor of the hospital.

### BOOKS RECEIVED.

Religious Tract Society.

"Faith Healing." By A. Schofield, M.D.

Raithly, Lawrence, and Co.

"Obstetric Hints." By Maitland Coffin.

The Scientific Press (Limited).

"Outlines of Insanity." By F. Walmsley.

David Nutt.

"Interpretation of Disease," Part II. By H. C. Gillies, M.B.

Adam Brothers.

"Health Culture." By G. Jaeger, M.D.

Churchill.

"Handbook of Hygiene," 7th Edition. By G. Wilson, M.D.

### THE STUDENT OF MEDICINE.

[All communications for this Supplement should be addressed to The Editor of The Hospital, at 140, Strand, with the words, "Students' Column," written in the left hand top corner of the envelope.]

### APPOINTMENTS.

Barendt, Frank H., M.D.Lond., F.R.C.S.Eng., appointed Pathologist to the Royal Southern Hospital, Liverpool. Bulstrode, Herbert Timbrell, M.A., M.D.Cantab., L.R.C.P.Lond., M.R.C.S.Eng., appointed Medical Inspector to the Local Government Board. Croom, John Halliday, M.D., F.R.C.P., F.R.C.S. Edin., appointed Physician to the Edinburgh Maternity and Simpson Memorial Hospital. Drummond, Wm. Blackley, M.B., C.M.Edin., appointed Resident Physician to the Royal Edinburgh Hospital for Sick Children. Fenwick, W. Soltau, M.D. Lond., M.R.C.P., appointed Medical Tutor to the London Hospital. Frazer, E. E., M.R.C.S., L.R.C.P., L.S.A., appointed House Surgeon to the Royal United Hospital, Bath. Ince, W. H., Ph.D., Würzburg, Demonstrator of Chemistry in University College, Liverpool, appointed Demonstrator of Physics and Chemistry in the Medical School of St. Thomas's Hospital. Lucas, Albert, L.R.C.P.Lond., F.R.C.S.Eng., appointed Resident Surgical Officer at the General Hospital, Birmingham. Robertson, Geo., M.B., C.M.Aberd., appointed Medical Officer to the Glenfoundland Lodge of Oddfellows, Insch, Aberdeenshire. Robertson, W. Ford, M.B., C.M.Edin., appointed Resident Physician to the Royal Edinburgh Hospital for Sick Children. Speechly, Harry Martindale, M.R.C.S.Eng., L.R.C.P.Lond., appointed House Surgeon to the London Hospital. Symonds, Horatio, F.R.C.S.Edin., of Oxford, appointed Honorary Consulting Surgeon to the Faringdon Cottage Hospital. Syrett, Frank, M.R.C.S.Eng., appointed Resident Medical Officer to the Children's Hospital, Newcastle-on-Tyne. Vernon, H., L.S.A., appointed Assistant Medical Superintendent of the Infirmary of the Parish of Paddington. Walsh, L. H., M.R.C.S., L.R.C.P., L.S.A., appointed Resident Medical Officer to the Royal United Hospital, Bath. Watson, J., M.B.Durh., F.R.C.S.Eng., L.R.C.P.Lond., appointed House Physician to the Wolverhampton and Staffordshire General Hospital. Williams, E. R., M.D., appointed Surgeon to H.M. Prison, Carmarthen. Sugden, H. C., M.R.C.S., L.R.C.P.Lond., L.S.A., has been appointed Senior House Surgeon at the Bury Dispensary Hospital.

### VACANCIES.

The following vacancies are announced this week, the amount of salary in each case being given after the name of the institution:

### Resident Medical Officers.

County Asylum, Shrewsbury, Junior Assistant Medical Officer—Salary, £100 per annum, &c.

Deaconesses' Institute and Hospital, Tottenham, House Surgeon—Salary, £90 per annum.

Leeds Friendly Societies' Medical Association, two Surgeons—Salary, £208 per annum, &c.

Leeds Public Dispensary, near Briggate, Junior Resident Medical Officer—Salary, £85 per annum.

Manchester Royal Eye Hospital, House Surgeon—Salary, £70 per annum, with board, &c.

Tottenham Hospital, First Resident House Surgeon—Salary £90 per annum.

Wrexham Infirmary and Dispensary, House Surgeon—Salary, £80 per annum, with board, &c.

York Retreat, Assistant Medical Officer, experienced in lunacy practice—Salary, £150 per annum, with board, &c.

### Non-Resident Medical Officers.

Leeds Friendly Societies' Medical Association, Assistant Surgeon—Salary, £120 per annum,

Royal United Hospital, Bath, Honorary Physician.

### The Hospital Advertiser.

### THE STUDENT OF MEDICINE—(*continued*).

### PASS LISTS.

**Royal College of Physicians and Surgeons, Edinburgh, and Faculty of Physicians and Surgeons, Glasgow.**

The quarterly examinations for the Triple Qualification in Edinburgh, took place in July with the following results:—

First Examination.—Of 73 candidates, the following 47 passed: Arthur Edward Clayton, Richard Wolfendale, Frederick Gustave Wooding Deane, John Alfred Finch, Joseph Davison Begley, Johanna Fleck Gilchrist, Isabella Hardie Curr, Samuel Nicolson, Annie Caroline Smith, Robert Lee Brown, Margaret Penelope Monro, James Wilson McBrearty, John Graham, Patrick Taaffe Finn, Alexandra Mary Campbell Geddes, Agnes Lillie Cousins, Annie Gillespie, Patrick Joseph Sheedy, Walter Wood Valance, Ernest Alexander Roland Laing, Arthur Wellesley Ball, Edward James McCarthy Morris (with honours), Gertrude Keith, (with honours), James Barry, Albert Lewis Levy, Mary Finch Nannetti, Denis Fitzgerald O'Kelly, Samuel James (with honours), Duncan Ritchie, Arthur George Kewley, Sarah Brown McMordie, Rosa Turner, Ella Cecilia Rawlinson, George Percival Searle, John Scott, Emily Frances Fitz Simons, Maurice Hickey Enright, Elizabeth Shaw Cotton, Frederick Algernon Molloy, Elizabeth Latto Ewan, Winifred Jane Pierce, Agnes Irene Sinclair Coghill, Davis Johnston Moncrieff Vallange, Andrew Smyth, Ernest Edward Scott, Joseph Galbraith, James Gallie, and Charles Seymour Langley.

The following candidates passed in the respective divisions:—

In Chemistry.—Mary Harriet Simson.

In Biology.—Flora Rosina Cartwright Wood, Mildred Jane Wallace, Ethel Louie Starmer, Walter Hulbert Cox.

In Biology and Physics.—John Gawler Murray.

Second Examination.—Of 50 candidates the following 38 passed: David Dickson Muir, Edwin Charles Hayes, Matthew Wilson, Armécide Azib Sakir, Henry William Vaughan, Edward Augustine Rowan, Alfred Arthur Hill, Alfred Vernon Crompton Holt, Arthur O'Leary, Florence Ullick O'Sullivan, Thomas Owen Jones, Hugh Gillies, Lydia Ann Leney, William Edward Vice, John Kilpatrick Brownlees, Robert Fairweather, Florence Ada Holt, Elizabeth Marianne Erskine, Lillias Jane Thomson, Michael Breen, Robert Lee Brown, Henry Marmion, Charlotte Rhoda Hodgins, Eleanor Agnes Montgomery, Ella Stewart Caldwell, Mary Buchanan Lee, Jessie Ballard Ferguson, Frederick Smith, Augustin Benjamin Ryan, Francis Albert Hadden, John William Stoker, Walter Kirkby, Lewis Tyrer Jones, Beatrice Ritchie, Mary Janet Dodds, William Shaw, Charles Edward Gooderson Bateman, and Robert James Black.

The following candidates passed in the respective divisions:—

In Anatomy and Physiology.—John Athol McIlroy.

In Physiology.—Charles Wilfred Lawson.

In Materia Medica.—Cyril Purdon, James Alfred Meeke, Charles Alfred Carmaet Jones, George Henry Monkhouse, and John Barnard Voortman.

Final Examination.—Of 103 candidates the following 59 passed, and were admitted L.R.C.P. and S.E., and L.F.P. and S.G.: William Allan Lloyd-Davies, William Wilson, Jean Fraser Robertson, Frederick William Pogson, Patrick Noonan, Alice Marion Umpherston, George Henry Peake, Edgar Francis Eardley Baines, Charles George Rae, Elsie Maud Inglis, Percy Thomas Tolputt, Alfred Eugène Berry, John Marie Ratnayeke, Donald Keith McDowell, Fairgray Christie, John Augustus Bouck, John Samuel Martin, Haydn Brown, George Wayland Ancrum, William Johnson Langley, Edward Joseph Gilleran, Francis Cubbon Rogers, Robert Roberts Stitt, Daniel Joseph Murphy, John Wallace Collett, Alexander Jamieson Meikle, David Donald, Alfred Gerrard Ginders, William Moody, Charles Albert Smith, Arthur James Thomas, Servaas De Kock, James Alexander Harbison, Martin Lavan, Thomas Bennett, Henry Edwin Connor, Albert Norman, Edgar Christian Wynn Hughes-Games, William Barwise, Augustin Benjamin Ryan, John Jordan Harvey, Andrew John McNickle, Thomas Francis Devane, Thomas Francis Roche, William Brough Arthur, Thomas James Selby, John Fallon Calahan, Richard Joseph Bodkin, Samuel Walter Pitcher, Andrew Fernie, Hugh Barker Williams, Percy Edgar Overton, George Thomas Cooke Adams, Adam Dryden, Samuel Gawn, Charles Ryall, James Levell Benson, James Aloysius Walsh, and Herbert Plowden Weston.

The following candidates passed in the respective divisions:—

In Medicine and Therapeutics.—Irvin Baldwin, Herbert Arthur Edgar Noble, Henry Alfred Jones, and James Louis Scott Sherlock.

In Surgery and Surgical Anatomy.—Gerald Herbert Johnston, William Morris Keal, Edith Grace Collett, and George Herbert Kemp Ross.

In Midwifery and Medical Jurisprudence.—Norman Gladstone Douglas, Edward McClure Campbell, Charles Henry Hibbert, Thomas Dawson, Owen Gilmore, James Henry Dewhurst Stephenson, Gerald Herbert Johnston, Alfred Arthur Hill, Herbert Arthur Edgar Noble, Thomas Harcourt Lawrie, Thomas Lawson, Lamont Paterson, Robert Richard Hughes, Alastair Robertson, James Louis Scott Sherlock, Archibald Houghton Brown, and George Herbert Kemp Ross.

